# Adverse effects associated with favipiravir in patients with COVID-19 pneumonia: a retrospective study

**DOI:** 10.1590/1516-3180.2021.0489.R1.13082021

**Published:** 2022-04-02

**Authors:** Figen Öztürk Ergür, Murat Yıldız, Melahat Uzel Şener, Suna Kavurgacı, Ayperi Ozturk

**Affiliations:** I MD. Physician, Pulmonary Medicine Department, Health Sciences University Faculty of Medicine, Atatürk Chest Diseases and Thoracic Surgery Training and Research Hospital, Ankara, Turkey.; II MD. Physician, Pulmonary Medicine Department, Health Sciences University Faculty of Medicine, Atatürk Chest Diseases and Thoracic Surgery Training and Research Hospital, Ankara, Turkey.; III MD. Physician, Interventional Pulmonology Department, Health Sciences University Faculty of Medicine, Atatürk Chest Diseases and Thoracic Surgery Training and Research Hospital, Ankara, Turkey.; IV MD. Physician, Pulmonary Medicine Department, Health Sciences University Faculty of Medicine, Atatürk Chest Diseases and Thoracic Surgery Training and Research Hospital, Ankara, Turkey.; V MD, PhD. Associate Professor, Interventional Pulmonology Department, Health Sciences University Faculty of Medicine, Atatürk Chest Diseases and Thoracic Surgery Training and Research Hospital, Ankara, Turkey. Interventional Pulmonology Department Health Sciences University Faculty of Medicine, Atatürk Chest Diseases and Thoracic Surgery Training and Research Hospital Ankara Turkey

**Keywords:** Favipiravir [supplementary concept], COVID-19, Pneumonia, Coronavirus disease 2019, Adverse effect, Avigan

## Abstract

**BACKGROUND:**

Favipiravir is generally used in treating coronavirus disease 2019 (COVID-19) pneumonia in Turkey.

**OBJECTIVE:**

To determine the side effects of favipiravir and whether it is a good treatment option.

**DESIGN AND SETTING:**

Retrospective study conducted in Atatürk Chest Diseases and Chest Surgery Training and Research Hospital, Ankara, Turkey.

**METHODS:**

357 patients who completed favipiravir treatment at the recommended dose were included. 37 patients with drug side effects and 320 patients without drug side effects were examined in two groups.

**RESULTS:**

Side effects were observed in 37 (10.36%) out of 357 patients using favipiravir. The most common side effect was liver dysfunction, in 26 (7.28%) of the patients. The following other side effects were also observed: diarrhea (1.4%), nausea (0.84%), abdominal pain (0.28%) and thrombocytopenia (0.28%). One patient (0.28%) presented both increased transaminases and nausea.

**CONCLUSION:**

In this study, it was determined that favipiravir may constitute an alternative for treating COVID-19 pneumonia given that its side effects are generally well tolerated and not serious.

## INTRODUCTION

Since the first appearance of coronavirus disease 2019 (COVID-19) in Wuhan, China, in December 2019, this disease has evolved into a global pandemic.^1^ It is associated with a wide clinical spectrum of conditions, ranging from asymptomatic disease to fatal pneumonia.^2^ Treatment protocols remain limited to guideline recommendations and clinical studies. No specific treatment or prophylaxis has been introduced for use.

Favipiravir is a purine analogue and ribonucleic acid (RNA)-dependent polymerase inhibitor that is used for influenza treatment in Japan. It has shown efficacy against many RNA viruses, including Ebola, neurovirus and *Enterovirus*.^3^ It is generally considered safe, with good tolerability, low side-effect potential and a half-life of five hours. The reported side effects include diarrhea, elevated transaminase levels, hyperuricemia and neutropenia.^4^

## OBJECTIVE

In this study, we aimed to assess the side effects of favipiravir treatment among patients diagnosed with COVID-19 pneumonia, in order to discuss its role as a therapeutic option.

## METHODS:

This retrospective study was conducted in Atatürk Chest Diseases and Chest Surgery Training and Research Hospital, Ankara, Turkey. The study included patients admitted to our hospital and hospitalized due to confirmed COVID-19, between September 1, 2020, and October 1, 2020. COVID-19 was diagnosed using the reverse-transcription polymerase chain reaction (RT-PCR) test. In this retrospective study, data on clinical characteristics, along with laboratory and chest computed tomography (CT) findings, were retrieved from digital databases and patient files. Patients > 18 years of age were deemed eligible if complete laboratory and chest CT data for them were available, along with a COVID-19 diagnosis via real-time RT-PCR; or if they showed highly suspicious disease based on clinical-radiological findings, despite RT-PCR negativity.

In accordance with the treatment guidelines for adult COVID-19 patients endorsed by the Turkish Ministry of Health, these patients received a loading oral favipiravir dose of 1600 mg twice daily, followed by a maintenance dose of 600 mg twice daily, for a total duration of 5 to 10 days.^5^

Demographic data, underlying conditions, clinical signs and symptoms, laboratory and radiological findings, oxygen support requirement, supportive treatments and side effects arising during favipiravir treatment were recorded. Side effects were defined as elevated transaminases, gastrointestinal symptoms (nausea, diarrhea or abdominal pain), high blood sugar or thrombocytopenia. In addition, a few of the patients also had elevated baseline transaminases, but they were not excluded because transaminase levels could also be raised due to the disease itself.

The local ethics committee approved this study (date: Oct 8, 2020; number: 696).

### Statistical analysis

Statistical analysis was performed using SPSS 25.0 (Statistical Package for the Social Sciences for Windows, Inc.; Chicago, Illinois, United States). The compatibility of the data to normal distribution was investigated by means of the Kolmogorov-Smirnov test. Data showing the characteristics of continuous variables were expressed as the mean ± standard deviation or median (minimum-maximum); and categorical data as the number and percentage (%). Independent groups were compared using Student’s t test or the Mann-Whitney U test. The relationships between variables were evaluated using Pearson’s correlation analysis. Categorical variables were compared using the chi-square test. P values < 0.05 were considered statistically significant.

## RESULTS

Overall, 357 patients who completed the recommended favipiravir regimen were included. The patients were divided between those who did and those who did not experience drug side effects (n = 37 and n = 320, respectively). In both groups, males comprised the majority of cases (25/37, 67.6%; and 203/320, 63.4%, respectively). The mean ages in the two groups were comparable (62.88 ± 13.9 and 58.95 ± 13.08 years, respectively). Patients with favipiravir-related side effects had higher body mass index (BMI) (28.73 ± 4.51 versus 30.39 ± 4.76, P = 0.03) (Table 1).

**Table 1.** Demographic characteristics of the patients

	No side effects (n = 320)	With side effects (n = 37)	P-value
**Age, years, n ± SD**	62.88 ± 13.9	58.95 ± 13.08	0.102
**Gender, n (%)**			
Male	203 (63.4%)	25 (67.6%)	0.753
Female	117 (36.6%)	12 (32.4%)	
Favipiravir alone (N = 231), n (%)	212 (91.8%)	19 (8.2%)	0.1
Favipiravir + hq (N = 126), n (%)	108 (85.7%)	18 (14.3%)	
**BMI, kg/m^2^ ± SD**	28.73 ± 4.51	30.39 ± 4.76	**0.036**
**Comorbidities, n (%)**			
Hypertension	141 (44.1%)	8 (21.6%)	0.014
DM	96 (30%)	7 (18.9%)	0.224
CAD	57 (17.8%)	4 (10.8%)	0.401
CHF	19 (6%)	1 (2.7%)	0.707
COPD	49 (15.3%)	2 (5.4%)	0.167
Asthma	22 (6.9%)	3 (8.1%)	0.734
Cancer	32 (10%)	4 (10.8%)	0.778
CRF	13 (4.1%)	–	0.377
ILD	1 (0.3%)	1 (2.7%)	0.198
Rheumatological	9 (2.8%)	1 (2.7%)	ns
**Medications administered, n (%)**			
OAD	63 (19.7%)	2 (5.4%)	0.057
ACEI/ARB	77 (24.1%)	3 (8.1%)	**0.045**
Betablocker	49 (15.3%)	3 (8.1%)	0.352
Antiaggregant/coagulant	38 (11.9%)	4 (10.8%)	ns
**Blood parameters**			
WBC (×10^9^/l), mean ± SD	7780.16 ± 4839.21	8188.61 ± 4355.72	0.628
Lymphocytes (×10^9^/l), median (range)	1030 (128-44990)	1350 (300-10920)	**0.016**
Platelets (×10^9^/l), mean ± SD	230534.38 ± 104771.79	250837.84 ± 116872.8	0.271
Neutrophil/lymphocyte ratio, median (range)	4.71 (0.12-79.92)	4.46 (0.08-23.44)	0.237
AST(IU/l), median (range)	34 (8-262)	46 (20-332)	**0.012**
ALT(IU/l), median (range)	26 (7-258)	35 (9-348)	**0.016**
CRP (mg/dl), median (range)	86 (1-493)	84.62 (2.5-395.74)	0.811
D-dimer (μg/l), median (range)	790 (156-80000)	780 (200-12430)	0.347
Ferritin (ml/ng), median (range)	320.5 (15-1650)	536.6 (41-1650)	**0.03**
Creatinine (mg/dl), mean ± SD	1.12 ± 0.52	0.94 ± 0.25	**0.04**
SO_2_ (%), mean ± SD	87.52 ± 8.77	88.22 ± 6.36	0.64
**CT findings, n (%)**			
Normal	8 (3.2%)	1 (2.9%)	ns
Frosted glass	232 (92.1%)	30 (88. %2)	0.505
Consolidation	87 (34.5%)	19 (55.9%)	**0.026**
One sided	93 (29.1%)	15 (40.5%)	0.252
Double-sided	190 (59.4(%)	20 (54.1%)	0.252
Favipiravir usage time (days), n ± SD	6.97 ± 2.38	7.73 ± 2.63	0.072
**Days of hospitalization, n ± SD**	7.13 ± 4.27	10.78 ± 5.62	**< 0.001**
**Intensive care transportation, n (%)**	19 (5.9%)	4 (10.8%)	0.279
**Need for advanced oxygen therapy, n (%)**	37 (11.6%)	4 (10.8%)	ns
**Need for advanced medical treatment, n (%)**	47 (14.7%)	8 (21.6%)	0.387
**Exitus, n (%)**	37 (11.6%)	4 (10.8%)	ns

WBC: White blood cell count, BMI = body mass index; DM = diabetes mellitus; CAD = coronary artery disease; CHF = congestive heart failure; COPD = chronic obstructive pulmonary disease; CRF = chronic renal failure; ILD = interstitial lung disease; OAD = oral antidiabetics; ACEI/ARB = angiotensin-converting enzyme inhibitor/angiotensin-receptor blocker; AST = aspartate aminotransferase; ALT = alanine aminotransferase; CRP = C-reactive protein; SO_2_ = oxygen saturation; ns = non-significant; SD = standard deviation, hq = hydroxychloroquine.

The most common comorbidities in both groups were hypertension and diabetes mellitus (DM). The most frequently used medications were oral anti-diabetics, angiotensin-converting enzyme inhibitors (ACEIs), angiotensin-receptor blockers (ARBs) and antiaggregating/anticoagulant agents. Patients without favipiravir-related side effects were more likely to be on ARB treatment (3/77; P = 0.04). Table 1 compares the demographic data, clinical characteristics and radiological findings in the two groups.

The duration of favipiravir treatment was comparable in the two groups. However, patients with favipiravir-related side effects had a significantly longer hospital stay (7.13 ± 4.2 versus 10.78 ± 5.62 days; P < 0.001) and were twice as likely to require intensive care unit admission (5.9% versus 10.8%; P = 0.279). While there were more patients requiring advanced medical treatment among those with favipiravir-related side effects, the difference was insignificant (21.6% versus 14.7%; P = 0.38) (Table 1). Among the 357 patients who received favipiravir, 37 (10.36%) had side effects. Among the 231 patients who received favipiravir alone, 19 (8.2%) had side effects, while among the 126 patients who received hydroxychloroquine with favipiravir, 18 (14.3%) exhibited side effects. The incidence of side effects did not differ significantly between these two groups (P = 0.1).

**Figure 1.**
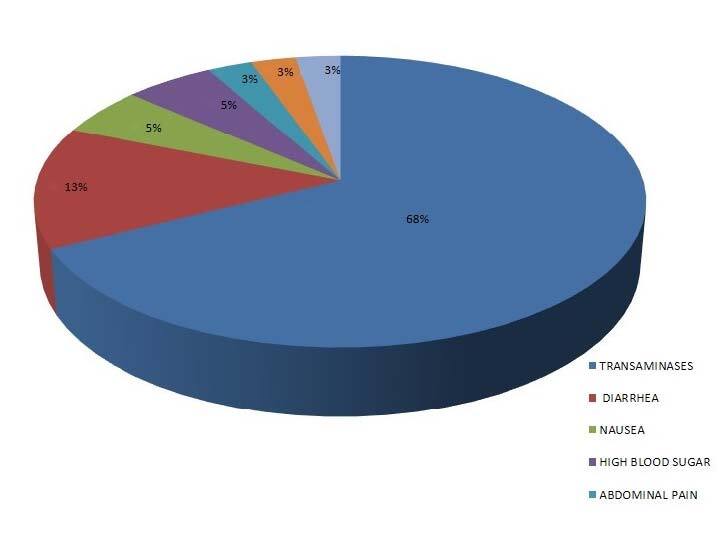
Distribution of side effects.

The most frequent drug-related adverse effect (elevated transaminase levels) occurred in 26 patients (7.28%, 26/357); in all cases, this did not require treatment discontinuation and was resolved through supportive therapy. Other adverse effects comprised diarrhea (1.4%), nausea (0.84%), abdominal pain (0.28%) and thrombocytopenia (0.28%). One patient showed elevated transaminases in conjunction with nausea (Table 2; Figure 1).

**Table 2.** Side effects seen with favipiravir

Patients using favipiravir	357
Patients with drug side effects, n (%)	37 (10.4%)
**Side effects, n (%)**	
Elevated transaminases	25 (7%)
Diarrhea	5 (1.4%)
Nausea	2 (0.56%)
High blood sugar	2 (0.56%)
Abdominal pain	1 (0.28%)
Thrombocytopenia	1 (0.28%)
Nausea + elevated transaminases	1 (0.28%)

A correlation analysis on determinants of favipiravir-related side effects showed that there were positive correlations with BMI and elevated baseline transaminase and ferritin levels; and negative correlations with elevated creatinine and ARB/ACEI use. However, all of these correlations were weak (r ≤ 0.2) (Table 3).

**Table 3.** Relationship between favipiravir side effects and clinical-laboratory-radiological features

Clinical-laboratory-radiological features	Favipiravir side effects	
	r (Pearson’s correlation coefficient)	P-value
Body mass index	0.111	**0.036**
Lymphocyte count	0.05	0.344
Aspartate aminotransferase	0.212	**< 0.001**
Alanine aminotransferase	0.216	**< 0.001**
Ferritin	0.144	**0.009**
Creatinine	-0.109	**0.04**
Consolidation in thorax tomography	0.143	**0.015**
Angiotensin-converting enzyme inhibitors/angiotensin-receptor blockers	-0.117	**0.027**

## DISCUSSION

One major challenge with COVID-19 relates to the absence of reliable evidence regarding therapeutic options. Furthermore, urgent therapeutic needs have resulted in the use of empirical treatments and other agents that were previously investigated for treatment of other coronaviruses. Several drugs, such as chloroquine, umifenovir, remdesivir and favipiravir, are being used for COVID-19 treatments in many countries, including Iran, Japan and China.^6,7,8,9^

Favipiravir, an antiviral agent in the nucleotide-analogue class, has been approved for influenza treatment in Japan. Its activity profile against influenza, Ebola and many other RNA viruses has been established as consisting of prevention of viral replication via inhibition of viral RNA polymerase.^10^ It is used as a pro-drug, with 94% bioavailability, 54% protein binding and low volume of distribution. Cmax (maximum concentration) is reached within two hours following a single dose, while both Tmax (maximum concentration time) and half-life increase after multiple doses. It has a half-life of 2.5 to 5 hours and is metabolized via rapid renal elimination after hydroxylation, mainly through the action of aldehyde oxidase and marginally through xanthine oxidase. It exhibits dose-and time-dependent pharmacokinetic effects. While not metabolized by the cytochrome P450 (CYP) system, favipiravir inhibits one of the system’s components (CYP 2C8). As such, caution is advised when it is co-administered with drugs metabolized by the CYP system.^11,12^

The dose of favipiravir administered varies based on indication. For COVID-19, higher doses are generally preferred.^13,14^ According to the national COVID-19 treatment guidelines issued by the Ministry of Health, a twice-daily dose of 1600 mg is recommended on the first day of treatment, followed by 600 mg, twice daily, for a total duration of 5 to 10 days, in patients with pneumonia and probable/definitive disease.^15^ All of our patients received this regimen. The most common side effects included elevated hepatic enzymes, gastrointestinal disorders, hyperuricemia and neutropenia.^5^

In a randomized Chinese study comparing favipiravir (at similar doses) with umifenovir (200 mg three times daily, for 10 days), 116 patients received favipiravir and 120 umifenovir. The recovery rates at day seven were comparable (P = 0.139), although the time to resolution of fever and coughing was shorter among favipiravir recipients. Side effects occurred in 31.9% of patients (n = 37) in the favipiravir group versus 23.33% (n = 28) in the umifenovir group (P = 0.14).^16^ In our study, 10.36% of the patients had side effects associated with favipiravir use. Transaminase elevation occurred in 8.62% of the patients in the favipiravir group (n = 10) versus 10% (n = 12) among umifenovir patients. Again, in the Chinese study, 13.79% of patients in the favipiravir group had increased serum uric acid levels, while this laboratory parameter was not examined in our study. Gastrointestinal reactions were reported in 13.79% of the patients, while occurring at a lower rate (2.24%) in the current study.

In a Japanese study,^17^ side effects were reported in 20% of the patients who received favipiravir in doses lower than those approved for COVID-19. Side effects were generally minor and, like in another study,^5^ included hyperuricemia (5%), diarrhea (5%), neutropenia and elevated hepatic enzymes (2%).^17^ In a review published in October 2020 that examined 32 studies registered at clinicaltrials.gov, elevated transaminase, eczema and pruritus, abdominal pain, nausea and diarrhea were reported in ≥ 1%, < 0.5%, 0.5%, 0.5 to 1% and ≥ 1% of the patients receiving favipiravir, respectively.^5^ In the current study, the most common adverse effect was elevated transaminases (7.28%), with no cases of allergic side effects, while gastrointestinal disorders (abdominal pain, diarrhea and nausea) were found in lower proportions.

Since favipiravir is metabolized and inhibited by aldehyde oxidase, serum concentrations should be monitored and dosing should be adjusted in those with hepatic impairment. Favipiravir, or its metabolites, has been detected in semen and breastmilk.^11,12^ Although favipiravir may have pharmacokinetic interactions with oseltamiver, its co-administration with acetaminophen in healthy volunteers resulted in excess exposure to acetaminophen. Meanwhile, as co-administration with theophylline may elevate blood drug concentrations, a concomitant increase in the risk of side effects occurs, thus requiring added caution during use of this combination.

In an interaction chart published by Liverpool Drug Interaction Group in March 2020, favipiravir does not have clinically significant interaction potential with ARBs/ACEIs.^18^ Therefore, the presence of more patients with ARB/ACEI use in our group without favipiravir-related side effects may simply have represented a coincidental finding, supportive of the absence of clinical interactions between favipiravir and ARBs/ACEIs.

Clinically significant interactions between favipiravir and hydroxychloroquine are unlikely. Favipiravir is mainly metabolized by aldehyde oxidase, while hydroxychloroquine is metabolized by the CYPs 2C8, 3A4 and 2Da. It is eliminated via urine, such that 3% of the administered dose is recovered within 24 hours. While favipiravir inhibits CYP 2C8, significant side effects are unlikely, given that hydroxychloroquine is metabolized through multiple pathways.^19^ This mechanism may explain the lack of difference between patients who received favipiravir alone and those who received favipiravir with hydroxychloroquine.

Favipiravir appears to be a good therapeutic option for treatment of COVID-19, as it can be administered orally and may also be given to symptomatic patients who do not require hospitalization. Like other antiviral agents, it may be recommendable to initiate favipiravir treatment soon after emergence of symptoms, given its ability to reduce viremia. This may certainly have some epidemiological implications in pandemics, such as in relation to COVID-19. Although side effects are generally well tolerated, laboratory parameters should be closely monitored.

The limitations of the present study were that it was conducted in a single center and had a retrospective design. This study was carried out in accordance with the recorded information only. Thus, it was not easy to reach conclusions regarding risk factors, given that it is possible that not all of them were considered because of the nature of retrospective cohorts.

## CONCLUSIONS

Favipiravir is a valuable drug for treatment of mild to moderately severe symptomatic COVID-19 patients. However, further randomized and controlled studies are warranted to provide more reassuring data for physicians regarding its use.
